# Long-term changes of Th17 and regulatory T cells in peripheral blood of dogs with spinal cord injury after intervertebral disc herniation

**DOI:** 10.1186/s12917-023-03647-8

**Published:** 2023-07-22

**Authors:** M. Wesolowski, P. Can, K. Warzecha, F. Freise, R. Carlson, J. Neßler, A. Tipold

**Affiliations:** 1 Department of Small Animal Medicine and Surgery, University of Veterinary Medicine, Hannover, Germany; 2 Department of Surgery, Faculty of Veterinary Medicine, University of Ankara, Ankara, Turkey; 3 Department of Biometry, Epidemiology and Information Processing, University of Veterinary Medicine, Hannover, Germany

**Keywords:** Lymphocytes, Canine, Th17, Treg, Flow cytometry, Spinal cord, Trauma

## Abstract

**Background:**

Intervertebral disc herniation (IVDH) is one of the most common causes of spinal cord injury (SCI) in dogs. As a result of acute SCI, a complex inflammatory response occurs in the spinal cord. Th17 cells (Th17) produce pro-inflammatory cytokines, while regulatory T cells (Treg) have opposite effects producing anti-inflammatory cytokines. Therefore, the aim of this study was to determine whether Th17- and Treg cells are involved in the pathogenesis of SCI or whether cellular changes occur due to coexisting inflammatory diseases. We hypothesized that chronic alterations in the Th17/Treg ratio are associated with a worse outcome after SCI.

**Methods:**

Twenty-six paretic or plegic dogs with IVDH with and without coexisting inflammatory disease were investigated in the acute stage of the disease and after recovery of SCI. In addition, a healthy control group was included (*n* = 14). Quantification of Th17 and Treg cells, from peripheral blood samples, was performed by multicolor flow cytometry and IL17 was measured using an enzyme-linked immunosorbent assay (ELISA).

**Results:**

After recovery significantly higher levels of Th17 (*p* = 0.0265) and Treg cells (*p* = 0.00025) were detected compared to acute IVDH but Th17/Treg ratio did not differ significantly. Recovered dogs and the control group did not differ significantly from each other. No association between an imbalance in the ratio and neurologic severity or underlying inflammatory diseases was found.

**Conclusion:**

This study demonstrated that altered Th17 and Treg levels in peripheral blood are altered in the acute stage of IVDH, preexisting inflammatory diseases seem not to influence these cell populations. Th17 and Treg cells could be considered when evaluating new treatment strategies for SCI.

**Supplementary Information:**

The online version contains supplementary material available at 10.1186/s12917-023-03647-8.

## Background

Spinal cord injuries (SCI) in dogs are often caused by intervertebral disc herniation (IVDH) [1, 2]. Commonly occurring signs are pain and paralysis of varying degrees [3, 4]. The recovery time takes several months, depending on the severity and extent of the pathophysiological processes in the spinal cord [5–7]. The inflammatory responses in the spinal cord are complex and have not been fully understood [8–10]. Animal models suggest that T lymphocytes have an important role in these pathophysiological processes [11, 12].

This study focuses on Th17 cells (Th17) and regulatory T cells (Treg) in dogs with SCI due to IVDH. Th17 cells are particularly known to fight extracellular bacteria and fungi [13, 14]. They preferentially produce the proinflammatory cytokine interleukin 17 (IL17), which activates neutrophilic granulocytes and recruits them to sites of infection [14]. Treg cells are important for immune tolerance and regulation of immune responses [15–17]. They produce anti-inflammatory cytokines, especially interleukin 10 (IL10) and transforming growth factor ß (TGFß), can prevent Th17 cell differentiation and counteract excessive immune responses [17, 18]. Basically, there is a close reciprocal relationship between Th17 and Treg cells [19–21]. A shift in the balance between Th17 and Treg cells in favor of proinflammatory Th17 cells has been detected in several autoimmune diseases, including systemic lupus erythematosus, inflammatory bowel disease, and chronic obstructive pulmonary disease [22–25]. Furthermore, an imbalance in Th17/Treg ratio has been identified in humans with chronic low back pain [26]. The results of several studies suggest the involvement of Th17 cells in the pathogenesis of IVDH [27–29]. Low Th17 levels as well as high IL17 levels were detected in dogs with acute IVDH compared to a healthy control population [28]. Up until today, the exact influence of Th17- as well as the interaction of Th17- and Treg cells in dogs with SCI is still not clear [28].

In the present study, measurements of Th17- and Treg cells were performed from peripheral blood samples in dogs with IVDH in the acute stage of the disease and after healing of SCI, at a time when inflammatory reactions in the spinal cord were no longer expected. We compared Th17, Treg cells and the Th17/Treg ratio at both time points and investigated the influence of coexisting inflammatory/immunological diseases and neurological severity. The overall goal of this work was to detect whether altered Th17 and Treg cell numbers were caused by IVDH itself or by coincidentally underlying coexisting inflammatory/immunological diseases. We hypothesized, that an altered Th17/Treg ratio after recovery of SCI would indicate a chronic imbalance caused by an underlying disease that negatively affects the clinical course. This work will provide the basics to develop targeted therapeutic interventions that affect Th17 and Treg cells if the hypothesis can be confirmed.

## Material and methods

### Study population

Sixty-five client-owned dogs with acute clinical signs of IVDH that were treated at the Department of Small Animal Medicine and Surgery of the University of Veterinary Medicine Hannover were included in this study. Clinical examination and peripheral blood sampling were performed before magnetic resonance imaging (MRI) diagnosis and before decompressive surgery for IVDH treatment as well as after proposed recovery of SCI, as part of a final examination. The diagnoses were made between April 2020 and November 2020. The final examinations after recovery of SCI were performed between August 2021 and December 2021 in 34/65 patients (52.3%), as 13 of these dogs were dead, 15 owners did not consent to a follow-up examination, and three owners could not be contacted anymore. Of the dead dogs, 10/13 (76.9%) were euthanized due to IVDH.

The time between the diagnosis of IVDH and the final examination was 14 months on average (median: 14 months, range: 11—18 months).

The collected data included history, results of the general clinical examination, and the clinical neurological examination. Results of the clinical neurological examination were used to grade neurological severity according to Sharp & Wheeler (Table 1) [30]. Neurological severity before diagnosis and treatment was further divided into two broad categories: “paresis” and “plegia”. Peripheral blood samples were collected at both timepoints.

**Table 1:** Grading of clinical severity of intervertebral disc disease in the study population

**Grading according to Sharp and Wheeler **[1, 2]	**Acute stage of the disease (total number of dogs)**	**Final examination (total number of dogs)**
**0 = no signs of spinal hyperesthesia or paresis**	0	14
**1 = spinal hyperesthesia only**	1	0
**2 = ambulatory paresis**	9	10
**3 = non ambulatory paresis**	6	1
**4 = plegia with intact deep pain perception**	7	0
**5 = plegia with loss of deep pain perception**	3	1

Dogs with clinical signs of acute disease or newly emerging diseases at the time point of clinical recovery, currently applied immunosuppressive therapy, and pathological changes in the differential blood cell count, such as leukocytosis or lymphopenia, were excluded from this study. Technical problems in processing the blood samples and missing data at the acute stage of IVDH were further exclusion criteria.

### Blood samples

Blood samples were taken either from the Vena cephalica antebrachii or the Vena saphena. Two EDTA samples (sample 1: 0.5—1 ml, sample 2: 1.4—3.8 ml) and one serum sample (1 ml) were taken (Sarstedt AG & Co, Nürnbrecht, Germany). Sample 1 was used to obtain a differential blood cell count immediately after blood collection using an Advia2120i Hematology System (Siemens Healthcare, Eschborn, Germany). Identification of the absolute lymphocyte count was necessary for accurate calculation of Th17 and Treg cell count. Sample 2 was stored at room temperature, protected from light, and used within 24 h to analyze Th17- and Treg cells. Serum samples were collected for enzyme-linked immunosorbent assay (ELISA), centrifugated (2000 g, 10 °C, 4 min; Hettich microliter centrifuge, Landgraf Laborsysteme HLL GmbH, Langenhagen, Germany), and supernatant was frozen at -20 °C until further measurement.

The study was performed and approved in accordance with the guidelines of the Animal Welfare Committee of the Lower Saxony State Government (LAVES, Lower Saxony, Germany) and the national animal welfare regulations (animal experiment number: 33.8–42502-05-20A561). All owners were informed about the study and gave their informed consent for anonymized analysis and publication of the data.

### Determination of Th17 and regulatory T-cells using multicolor flow cytometry

The determination of Treg cells was performed following the protocol of Knueppel et al. [31]. For the identification and quantification of Th17 cells, the measurement procedures according to Knebel et al. [32] and Kämpe et al. [33] were used as a modification of the methods developed by Kol et al. [34].

#### Separation of mononuclear cells and magnetic cell separation

Peripheral blood mononuclear cells (PBMC) were isolated from EDTA blood samples by density gradient centrifugation. For this, Lympho 24 + Spin Medium (density: 1.072 g/ml; pluriSelect Life Science Worldwide, Leipzig, Germany) was used according to manufacturer’s instructions. CD8alpha + , CD11b + , and CD21 + were labeled with mouse anti dog CD8 alpha (1:11, pre-dilution 1:5 with staining buffer, Bio-Rad Laboratories, Inc., California, USA), mouse anti dog CD 11b (1:11, Bio-Rad Laboratories, Inc., Hercules, California, USA) and mouse anti dog CD21 (1:11, pre-dilution 1:100 with staining buffer, Bio-Rad Laboratories, Inc., Hercules, California, USA). Goat anti mouse IgG MicroBeads (MACS Miltenyi Biotec GmbH, Bergisch Gladbach, Germany) were added prior to magnetic cell separation. Using the autoMACS® Pro Separator (Miltenyi Biotec GmbH, Bergisch Gladbach, Germany), the unwanted antibody-labeled cells (CD8alpha + , CD11b + , CD21 +) were separated from the desired target cells (CD8alpha-, CD11b-, CD21- cells). The unlabeled target population (CD8alpha-, CD11b-, CD21-) was further processed. A second manual cell count was performed and the cell suspension was divided into two portions to further process the Treg- and Th17 cells separately.

#### Further processing and staining of regulatory T cells

Viobility TM 405/520 Fixable Dye (1:100, Miltenyi Biotec GmbH, Bergisch Gladbach, Germany Miltenyi), was added to the cell suspension to distinguish between live and apoptotic or dead cells in the flow cytometric analysis performed later. Cell suspension was blocked with Human TruStain FcX TM (1:20, BioLegend®, San Diego, California, USA) and divided into three portions: a native fraction, an isotype fraction, and a test fraction [31].

**Fig. 1:**
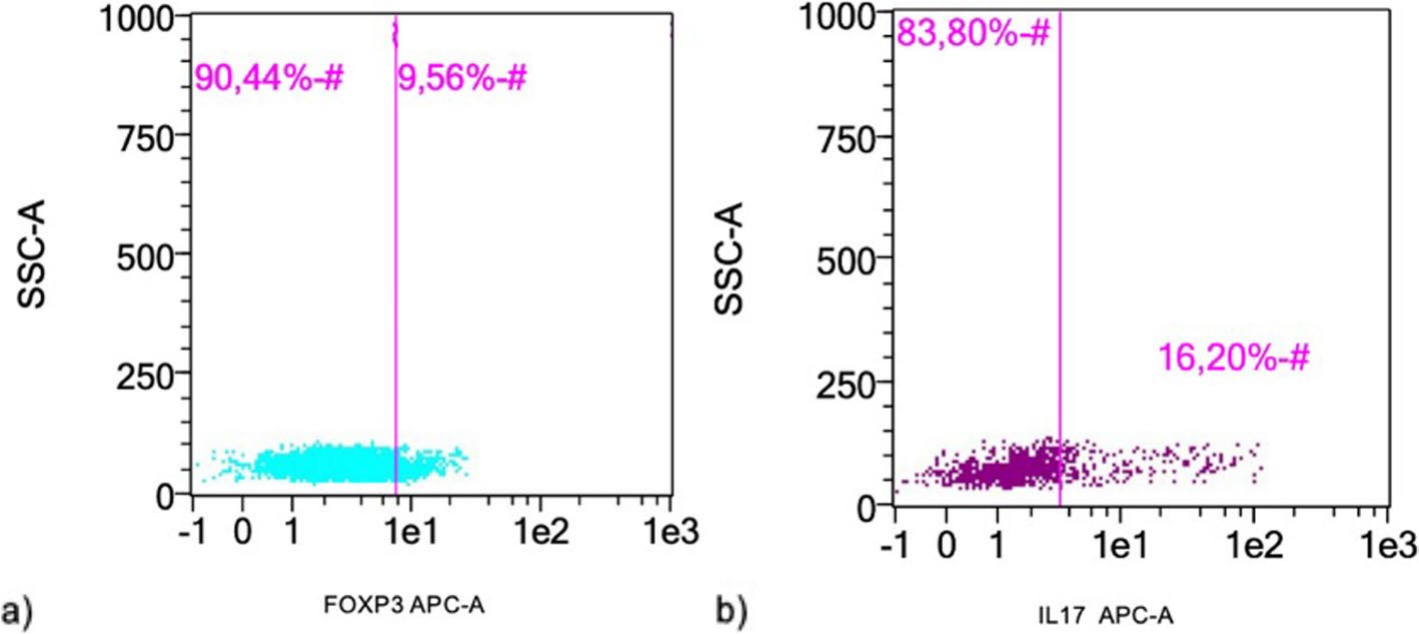
Illustration of a Treg and a Th17 cell analysis using flow cytometry Results of Treg and Th17 cell analysis of a dog after recovery of spinal cord injury using MACSQuant analysis software In the prior performed isotype controls, limits were set at approx. 2% of the cells [32, 34–36]. This limit was transferred to the test fractions (pink lines) in each case to detect the percentages of FOXP3 (a) or IL17 (b) positive cells, which are located to the right of the pink line Absolute cell numbers were subsequently calculated to make the analyses comparable a) The graphic displays the percentage of FOXP3 positive cells for detection of Treg cells. 9.56% FOXP3 positive cells were identified b) The graphic displays the percentage of IL17 positive cells for detection of Th17. 16.20% IL17 positive cells were identified. The results of a stimulated Th17 cell fraction are shown

In the following dyeing process mouse anti canine CD3 FITC (1:11, Bio-Rad Laboratories, Inc., California, USA), rat anti canine CD4 PE-Cy7 (1:11, affymetrix eBioscience, San Diego, California, USA) and mouse IgG1 PE (1:20, eBioscience ™, San Diego, California, USA) were added to the isotype fraction. Mouse anti canine CD3 FITC (1: 11, Bio-Rad Laboratories, Inc., Hercules, California, USA), rat anti canine CD4 PE-Cy7 (1:11, affymetrix eBioscience, San Diego, California, USA) and mouse anti canine CD25 PE (1:20, Bio-Rad Laboratories, Inc., Hercules, California, USA) were pipetted to the test fraction [31]. Afterwards, fixation and permeabilization of the cells was performed with the Foxp3/Transcription factor staining buffer set (eBioscience™ San Diego, California, USA). Intracellular staining was performed after blocking with Human TruStain FcX TM (1:20) using rat IgG2a kappa APC (1:11, eBioscience™, San Diego, California, USA) and rat anti canine FOXP3 APC (1:11, eBioscience™, San Diego, California, USA) was pipetted to the test tube. Cell fractions were resuspended in 200 µl FACS-staining buffer and subsequently flow cytometric determination of Treg cells with the MACSQuant®Analyzer 10 (Miltenyi Biotec GmbH, Bergisch Gladbach, Germany) was performed [31]. Flow cytometric results were analyzed with the MACSQuantifyTM software (Miltenyi Biotec GmbH, Bergisch Gladbach, Germany) (Fig. 1a).

#### Further processing and staining of Th17 cells

Further processing of Th17 cells included a process of cell regeneration and stimulation due to the physiologically low number of Th17 cells in blood [32, 34–36].

The pellet for Th17 isolation was resuspended in 400 µl thawed lymphocyte culture medium (RPMI Medium1640, Gibco™ life technologies limited, Carlsbad California USA) with 5% fetal bovine serum (Gibco®, Thermo Fisher Scientific, Carlsbad California, USA), 1% HEPES solution (Sigma-Aldrich®, Taufkirchen, Germany) and 1% PenStrep (100 U/ml Penicilin-G and 100 μg/ml Streptomycin; Sigma-Aldrich®, Taufkirchen, Germany), and was transferred into two wells of a BRAND plates® cellGradeTM 96 well plate (Brand, Wertheim, Germany). This was stored in the incubator overnight (37 °C, 5% CO2).

The stimulation medium was composed of lymphocyte medium, phorbol-12 myristate 13-acetate (1:100, PMA 25 ng/mL; Cayman Chemical Company, Ann Arbor, Michigan, USA), and ionomycin (1:10, 500 ng/mL; Sigma-Aldrich®, Taufkirchen, Germany).

The cells in the first well were resuspended in the stimulation medium, whereas the cells in the second well were resuspended in the lymphocyte culture medium alone. The BRAND plates® cellGradeTM 96 well plate incubated for three hours in the incubator (37 °C, 5% CO2). Subsequently, Brefeldin A (10 mg/mL, 1:100, Sigma-Aldrich®, Taufkirchen, Germany) was added to the stimulated and unstimulated wells and the cell plate was stored in the incubator for another three hours (37 °C, 5% CO2).

Viobility dye (1:100, ViobilityTM 405/452 Fixable Dye, Miltenyi Biotec GmbH) and Human TruStain FcXTM (1:20) were added to each well. The analysis included one native, one isotype, and one test cell fraction. Rat anti canine CD4 PE-Cy7 (1:11, eBioscience Inc., San Diego, California USA) and mouse anti dog CD3 FITC (1:11, BioRad Laboratories, Inc., San Diego, CA, USA) were added to the test and the isotype wells. For cell fixation, a flow cytometry fixation buffer (R&D Systems®, Minneapolis, Minnesota, USA) was used. After fixation, the cells were permeabilized using saponin buffer (0.03% saponine (Alfa Aesar GmbH & Co. KG, Karlsruhe, Germany) in FACS-staining-buffer)).

Subsequently, the intracellular dyeing was performed by using biotin mouse IgG1, k isotype Ctrl (eBioscience, San Diego, California, USA) for isotype control and IL-17A-Biotin, mouse monoclonal antibody (1:99 with saponin buffer; Dendritics SAS, Lyon, France) for test samples and streptavidin (50 µl, pre-dilution 1:100 in saponin buffer, BioLegend®, San Diego, Kalifornien, USA) was added to iso- and test wells. After resuspension of the cell fractions in 200 µl FACS staining buffer, flow cytometric determination of Th17 cells could be performed using MACSQuant®Analyzer 10 [27]. Subsequently, MACSQuantifyTM software was used for further analysis (Fig. 1b).

### IL17 ELISA

The IL17 ELISA was performed with serum samples according to the manufacturer’s instructions (MonELISA® Canine IL-17, mdbioproducts., St. Paul, USA).

The provided detection range was 25—1600 pg/ml and the ELISA was performed in compliance with the criteria described by Kämpe [28]. Repeatedly non-measurable samples were excluded from the study.

### Statistical analysis

Flow cytometric results were analyzed with the MACSQuantifyTM software (Miltenyi Biotec GmbH, Bergisch Gladbach, Germany). The absolute cell count was calculated by using Microsoft® Excel 2022 (Microsoft Corporation, Washington, USA). Microsoft® Excel 2022 was generally used for documentation of the measurement results. Statistical analyses were performed using SAS Enterprise Guide, version 7.15, and SAS® software version 9.4 (SAS Institute Inc., Cary, North Caroline, USA). Graphical plots were created with GraphPad Prism® version 9 (GraphPad Software Inc., La Jolla, California, USA). The Th17, Treg cell counts, and the Th17/Treg ratio were investigated at the onset of disease (“acute”) and after healing of SCI (“outcome”). The influence of two groups (with and without coexisting underlying inflammatory/immunological disease) and neurological severities (paresis and plegia) were analyzed individually and in combination with the timepoint “acute” and “outcome”. *P*-values ≤ 0.05 were considered significant.

Data were tested for normal distribution by Kolmogorov–Smirnov test. A linear mixed model with timepoint, underlying inflammatory disease and neurological severity as factors, including all interactions and autocorrelated errors for the timepoints was fitted to the logarithm of the cell counts as well as their ratio. The p-values in the post-hoc analysis were adjusted according to Tukey–Kramer. Furthermore, Bonferroni adjustment was used to determine unilateral *p*-values for the following tests:

- all dogs at time point “acute” compared to “outcome”.
- dogs without underlying inflammatory disease at the time point “acute” compared to “outcome”
- dogs with underlying inflammatory disease at the time point “acute” compared to “outcome”
- paretic dogs at the time point “acute” compared to “outcome”
- plegic dogs at the time point “acute” compared to “outcome”

## Results

### Study population

First examination was performed in 65 patients at the acute time of IVDH. Final examination was available in 34 patients (see above). The following dogs were additionally excluded from the study:

One dog because of acute clinical signs of disease and leukocytosis, one dog because of immunosuppressive treatment, three dogs due to technical problems in processing the blood samples, and three dogs because of missing data at the acute stage of IVDH. Consequently, the final study population consisted of 26 patients (see Additional file 1).

Seven dogs (26.9%) were female intact, four dogs (15.4%) were male intact, six dogs (23.1%) were female neutered, and nine dogs (34.6%) were male neutered. Average age at acute IVDH was six years (range 3—7 years). Average age at the final examination was seven years (range 4–12 years). Average weight was 14.7 kg (range 4.2—34.4 kg). 13 different breeds were represented including French bulldog (*n* = 6, 23.1%), mixed breed dog (*n* = 5, 19.2%), Dachshund (*n* = 3, 11.5%), Labrador Retriever (*n* = 2, 7.7%), Bolonka Zwetna (*n* = 2, 7.7%), and one dog (3.8%) of each of the following breeds: Pug dog, Maremmano, Yorkshire Terrier, Maltese, Havanese, German Shepherd, Jack Russel Terrier, Miniature Bull Terrier.

The healthy control group included 14 Beagles with a gender distribution of eight intact females (57.1%), four intact males (28.6%) and two males neutered (14.3%) with an average weight from 12.9 kg (range: 9.9—16.2 kg) (see Additional file 7).

Twenty three dogs (88.5%) were diagnosed with intervertebral disc extrusion (IVDE) and 3 dogs (11.5%) with acute non compressive nucleus pulposus extrusion (ANNPE). All cases with IVDE were treated surgically, while all cases with ANNPE were treated conservatively. The neurolocalization of IVDH in the acute stage was in 76.9% (*n* = 20) cases T3—L3, in 15.4% (*n* = 4) cases L4-S1, and in 3.8% (*n* = 1) cases C1—C5 and C6—Th2 (see Additional file 2).

Seven dogs (26.9%) had a coexisting immunological disease or chronic inflammatory comorbidity (Table 1). Of these, patients had food allergy (2/7, 28.6.%), atopia (3/7, 42.9%), or chronic bladder infection (2/7, 28.6%).

The following grading according to Sharp and Wheeler [1, 2] was available for the dogs (Table 1): grade 1 in 3.8% (*n* = 1), grade 2 in 34.6% (*n* = 9), grade 3 in 23.1% (*n* = 6), grade 4 in 26.9% (*n* = 7), grade 5 in 11.5% (*n* = 3) at the acute stage of the disease. Dogs in the acute stage of the disease were divided into two broad categories: “paresis” and “plegia”. Since only one dog was initially Sharp and Wheeler [1, 2] grade 1, it was placed in the group of dogs with “paresis” for statistical evaluation. Thus, dogs with (16 dogs, 61.5%) and without (10 dogs, 38.5%) spontaneous movement were grouped together. At the final examination the resulting grading for the dogs is as follow: grade 0 in 53.8% (*n* = 14), grade 2 in 38.5% (*n* = 10), grade 3 in 3.8% (*n* = 1), grade 5 in 3.8% (*n* = 1).

When comparing the individual courses of disease, 88.5% (*n* = 23) showed an improvement of at least one severity level and 11.5% (*n* = 3) remained the same (see Additional file 2).

### Determination of Th17- and regulatory T- cells and investigation of the Th17/Treg ratio

#### Investigation of Th17 cells in dogs with intervertebral disc herniation and examination of the effects of time point, severity of neurological deficits, and an underlying disease

**Fig. 2:**
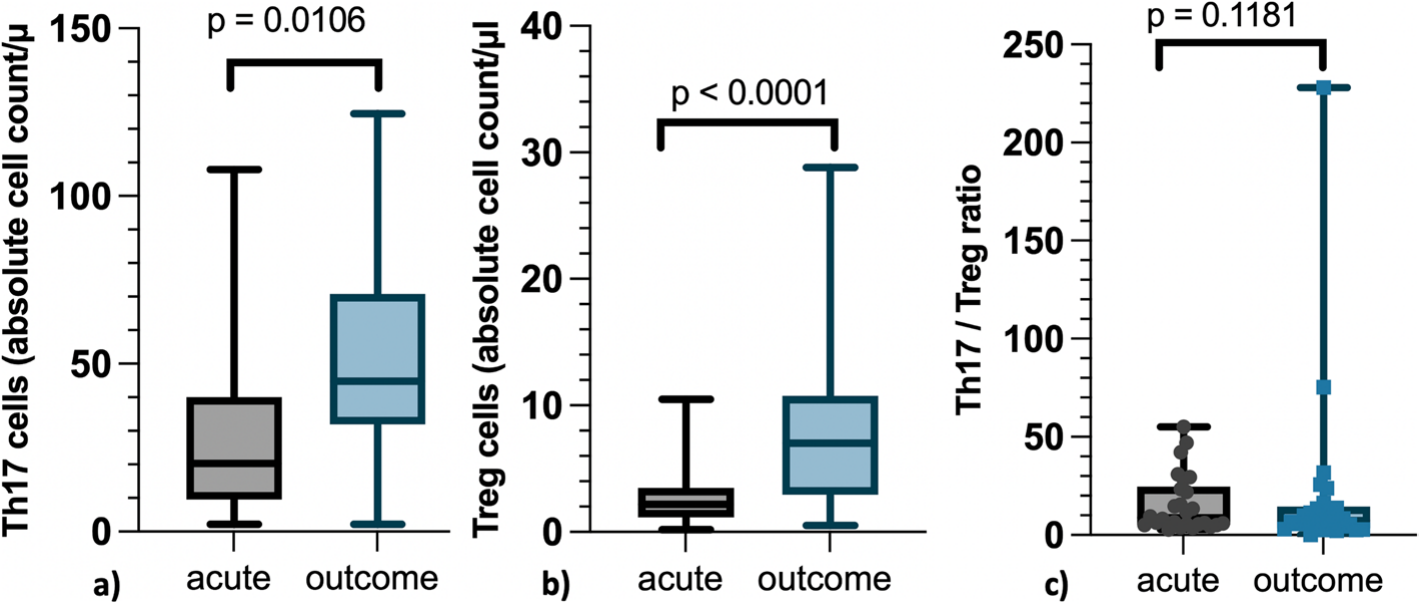
Th17-, Treg cells and Th17/Treg ratio before and after treatment of intervertebral disc herniation Absolute number of Th17 and Treg cells and the Th17/Treg cell ratio in the acute stage of disease (“acute”) and after recovery (“outcome”) are displayed The grey boxplot on the left represents the results in the acute stage of intervertebral disk herniation (IVDH) before treatment. The right-sided boxplot in light blue represents the results of the final examination, which took place on average 14 months after surgery. At both time points the same 26 dogs with IVDH were analyzed. Boxplots were used for the graphical representation. Each boxplot displays the 25% and 75% quantile and the median a) Absolute number of stimulated Th17 cells: Significant differences in Th17 cell levels between the time points “acute” and “outcome” (*p* = 0.0106) b) Absolute number of Treg cells: Significant differences in Treg cell levels between the time points “acute” and “outcome” (*p* < 0.0001) c) Th17/Treg ratio: No significant differences between the time points “acute” and “outcome” (*p* = 0.1181). Every measurement is shown as a single plot. At the time point “outcome” two ratios (75.2 and 227.9) differed strongly from the others

A statistically significant difference in Th17 cell count was detected between measurements in the acute phase and after recovery (*p* = 0.0106, Fig. 2a). There was a significant increase in Th17 cell count (*p* = 0.0265). The Th17 cell values after recovery did not differ significantly from the control population (*p* = 0.3203) (Fig. 5a).

**Fig. 3:**
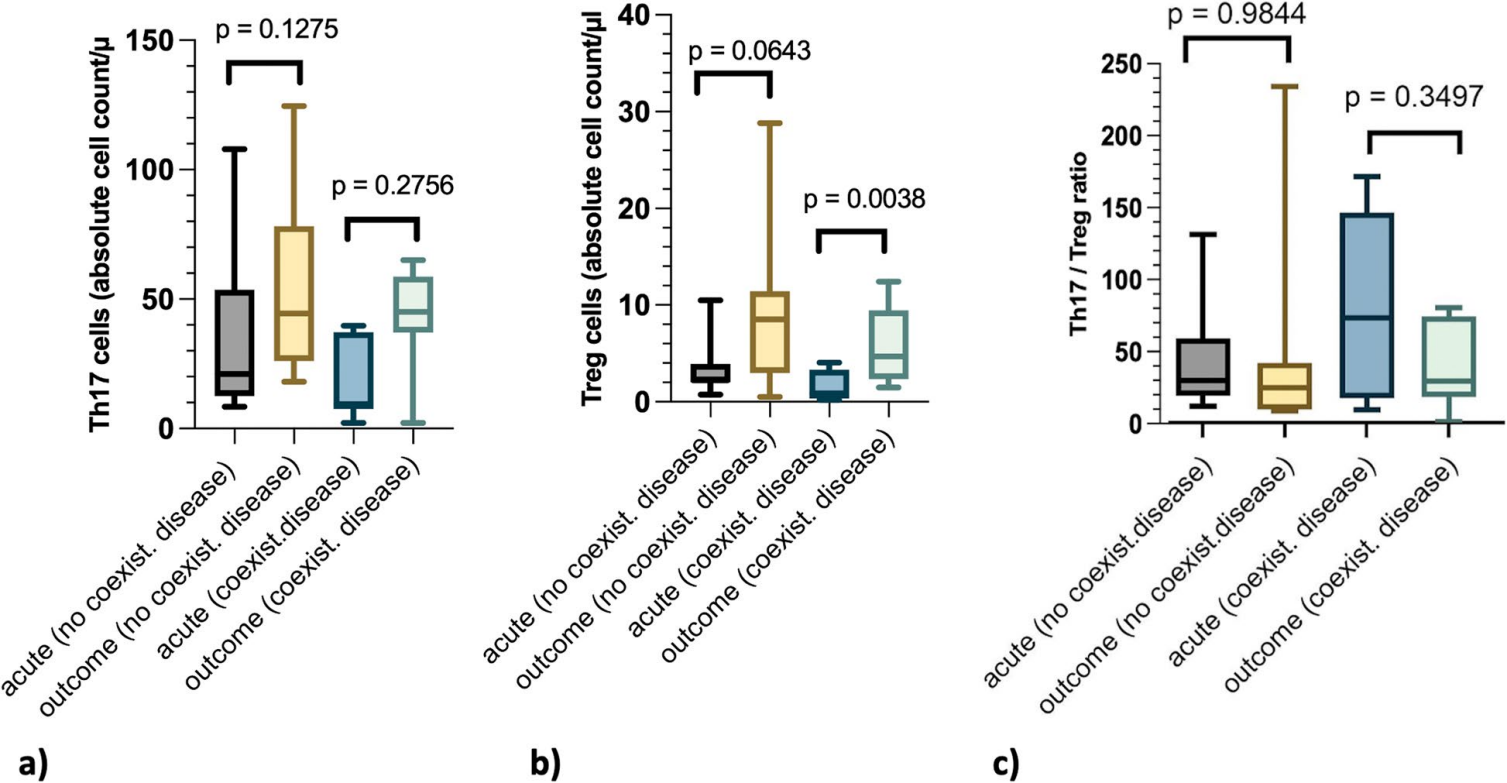
Investigation of the effect of an underlying disease in dogs with intervertebral disc herniation The effect of an underlying coexisting inflammatory/immunological disease on Th17-, Treg levels and the Th17/Treg ratio was investigated Each graphic is composed of 4 boxplots. The first (grey) and second (yellow) boxplot display data of dogs without a coexisting disease (“no coexist. disease”) at the time “acute” (first boxplot) and “outcome” (second boxplot). The third (light blue) and the fourth (green) one display data from dogs with a coexisting inflammatory disease (“coexist. disease”) at the time “acute” (third boxplot) and “outcome” (fourth boxplot) “acute” = acute stage of disease before treatment of IVDH “outcome” = after recovery, on average 14 months after disc surgery a) Th17 cells in absolute cell count/µl: No significant difference in dogs without a coexisting inflammatory disease between the time points “acute” and "outcome" (*p* = 0.1275). No significant difference in dogs with a coexisting inflammatory/ immunological disease between the time points “acute” and "outcome" (*p* = 0.2756) b) Treg cells in absolute cell count/µl: No significant difference in dogs without a coexisting inflammatory/immunological disease between the time points "acute" and "outcome" (p = 0.0643). Note the significant difference in dogs with a coexisting inflammatory/immunological disease between the time points "acute" and "outcome" (*p* = 0.0038) c) Th17/Treg ratio: No significant difference in dogs without a coexisting inflammatory/immunological disease between the time points "acute" and "outcome" (*p* = 09844). No significant difference in dogs with a coexisting inflammatory/immunological disease between the time points "acute" and "outcome" (p = 0.3497). The ratio 227.9 at the time point outcome was excluded from the graphic representation

Th17 cell count between dogs without underlying inflammatory/immunological disease and dogs with coexisting underlying inflammatory/immunological disease differed significantly when evaluating the effect individually without consideration of the time points “acute” and “outcome” (*p* = 0.0331). No statistical significant differences were detected at the “acute” (*p* = 0.2307) and “outcome” (*p* = 0.4509) time points and between the “acute” and “outcome” time points in dogs without (*p* = 0.1275, Fig. 3a) and with underlying immunological/inflammatory disease (*p* = 0.2756, Fig. 3a).

**Fig. 4:**
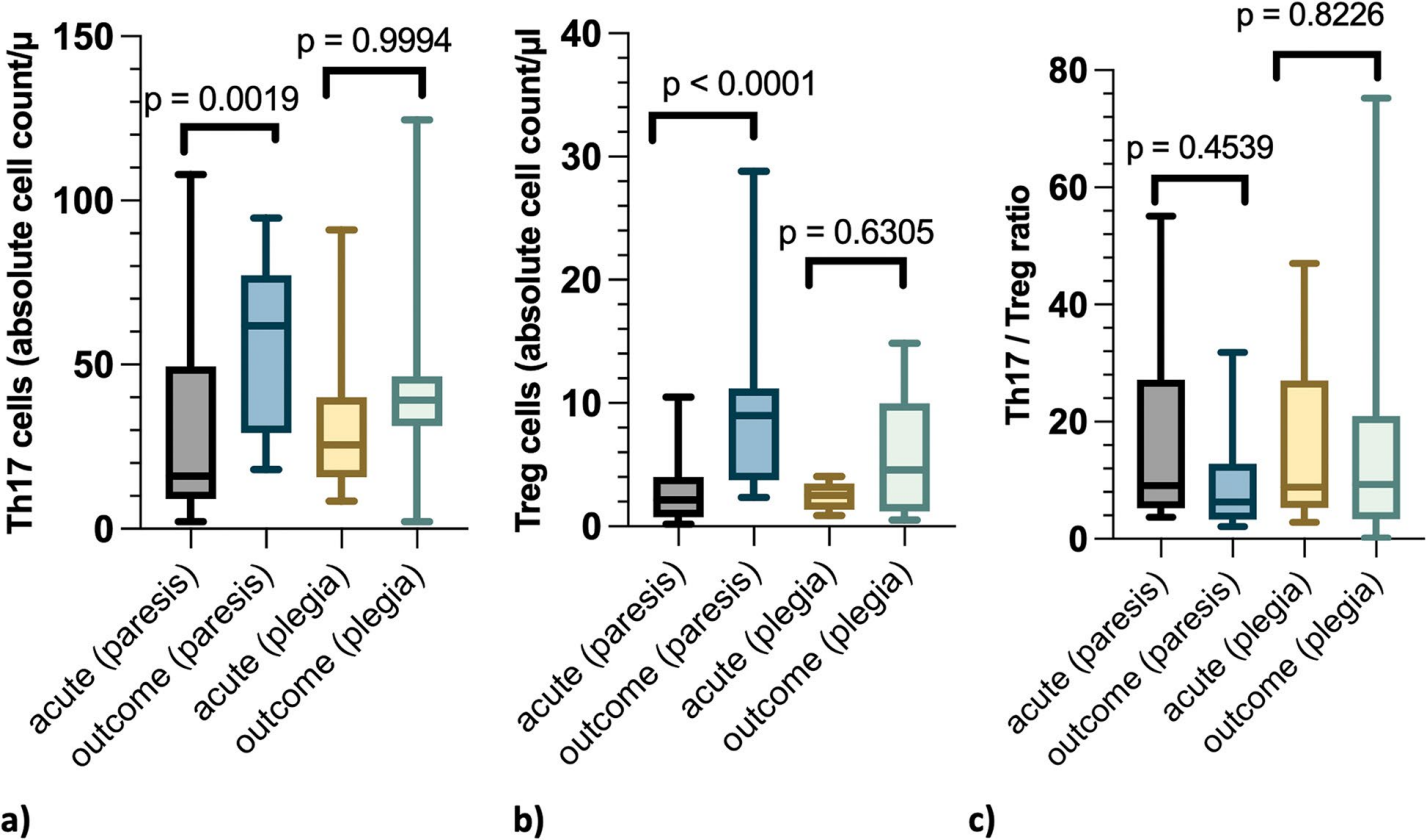
Investigation of the effect of neurological deficit severity in dogs with intervertebral disc herniation Comparison of Th17-, Treg cell levels and Th17/Treg ratio between dogs with paresis and plegia in the acute stage of the disease ("acute") and after recovery of intervertebral disc herniation ("outcome") Each graphic (a,b,c) is composed of four boxplots. The first (grey) and second (light blue) boxplot display data of dogs with paresis at the time points "acute" (first boxplot) and "outcome" (second boxplot). The third (yellow) and the fourth (green) one show data from dogs with plegia at the time "acute" (third boxplot) and "outcome" (fourth boxplot) "acute" = acute stage of disease before treatment of IVDH "outcome" = after recovery, on average 14 months after disc surgery a) Th17 cells in absolute cell count/µl: Significant difference in dogs with paresis between the time point “acute” and "outcome" (*p* = 0.0019). No significant difference in dogs with plegia between the time point “acute” and "outcome" (*p* = 0.9994) b) Treg cells in absolute cell count/µl: Significant difference in dogs with paresis between the time point "acute" and "outcome" (*p* < 0.0001). No significant difference in dogs with plegia between the time "acute" and "outcome" (*p* = 0.6305) c) Th17/Treg ratio: No significant differences neither in dogs with paresis between the time points “acute” and "outcome" (*p* = 0.4539) nor in dogs with plegia (*p* = 0.8226). The ratio 227.9 at the time point outcome was excluded from the graphic representation

Severity of neurological deficits after IVDH (paresis or plegia) had no statistically significant effect on Th17 cell count (*p* = 0.7911). There was no statistically significant difference in Th17 cell count between paretic and plegic dogs at both the “acute” (*p* = 0.4651) and “outcome” time point (*p* = 0.2606). However, in paretic dogs, there was a statistically significant difference between the acute phase and after recovery (*p* = 0.0019, Fig. 4a). Here, the Th17 cell count increased significantly (*p* = 0.001). In plegic dogs no statistically significant difference between these time points (*p* = 0.9994, Fig. 4a) occurred.

#### Investigation of regulatory T cells in dogs with intervertebral disc herniation and examination of the effects of time point, severity, and underlying disease

**Fig. 5:**
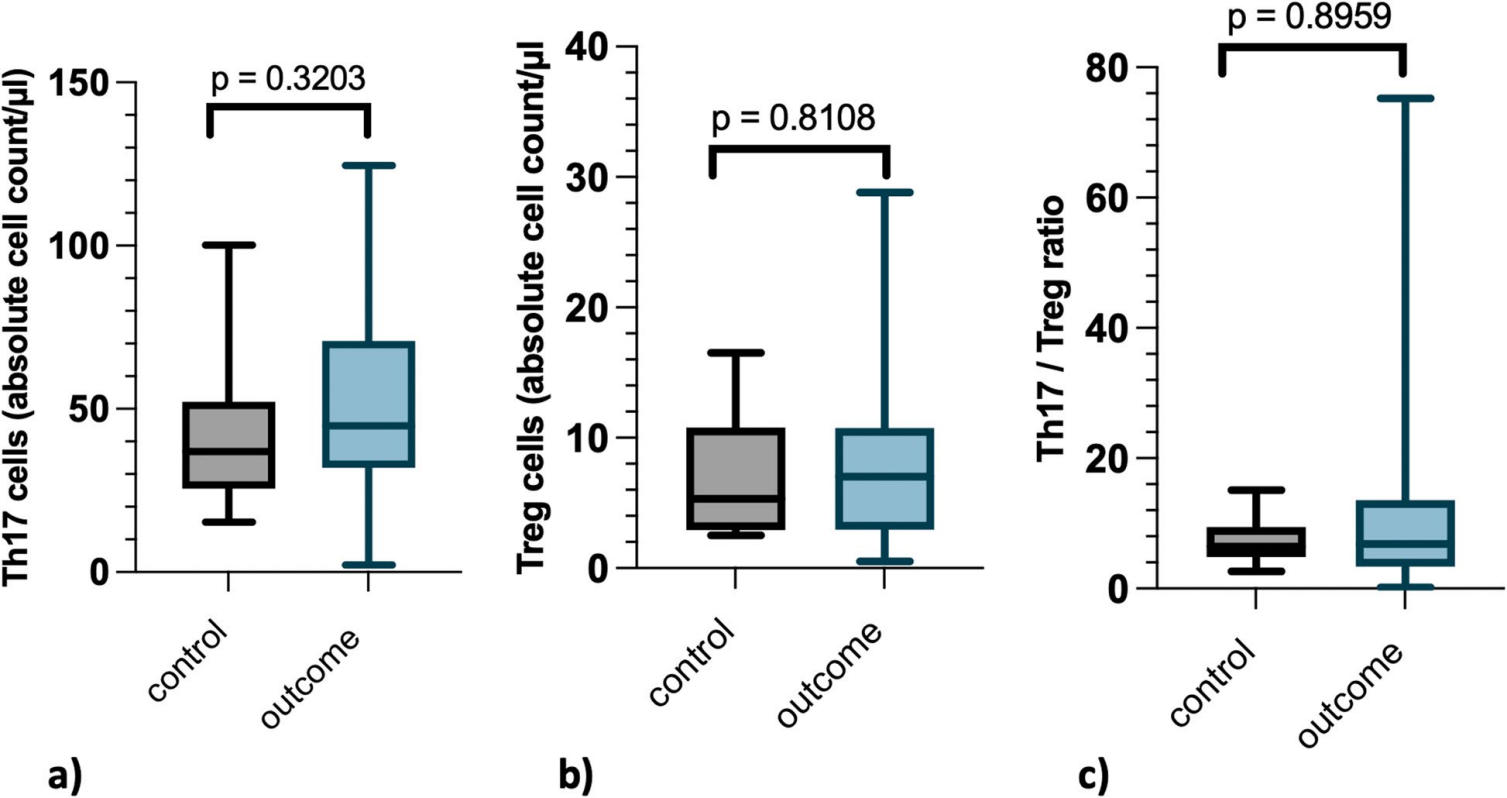
Comparison of cell counts in dogs after recovery from intervertebral disc herniation with a control group Comparison of Th17 cells, Treg cell levels and the Th17/Treg ratio from dogs after recovery ("outcome") from intervertebral disc herniation (IVDH) (*n* = 26) with a healthy control population ("control", *n* = 14). The left boxplot (grey) displays the data of the "control" group. The right boxplot (light blue) displays the data of the dogs after recovery from IVDH ("outcome") a) Th17 cells in absolute cell count/µl: No significant difference between "outcome" and "control" (*p* = 0.3203) b) Treg cells in absolute cell count/µl: No significant difference between "outcome" and "control" (*p* = 0.8108) c) Th17/Treg ratio: No significant difference between "outcome" and "control" (*p* = 0.8959). At the time point "outcome" the ratio 227.9 was excluded from this graphic.

A statistically significant increase (*p* = 0.000025) was found in the number of Treg cells between the acute phase and after recovery (*p* < 0.0001, Fig. 2b). Treg cell levels after recovery were not significantly different from the corresponding Treg cell levels of the control population (*p* = 0.8108) (Fig. 5b).

The presence of underlying inflammatory/immunological disease did not result in a statistically significant difference in the number of Treg cells (*p* = 0.1146). There were no statistically significant differences in the Treg cell count of dogs without and with underlying inflammatory/immunological disease at the "acute" (*p* = 0.0909) and "outcome" (*p* = 0.9997) time points.

While there was no significant difference between the "acute" and "outcome" time points in dogs without underlying inflammatory/immunological disease (*p* = 0.0643, Fig. 3b), there was a significant difference in the number of Treg cells between these time points in dogs with underlying disease (*p* = 0.0038, Fig. 3b). In unilateral testing, using a Bonferroni adjustment, there was a significant increase in Treg cell number between these time points in both groups of dogs without underlying disease (*p* = 0.03625) and with underlying disease (*p* = 0.00175).

Clinical severity of paresis or plegia had no significant effect on Treg cell counts (*p* = 0.9000).

At both, the acute stage (*p* = 0.2606) and after recovery (*p* = 0.3515), there was no significant difference in Treg cell number in paretic and plegic dogs.

However, a highly significant difference in Treg cell counts between the "acute" and "outcome" time points in paretic dogs (*p* < 0.0001, Fig. 4b) was found. Treg cell number increased significantly (*p* < 0.00025), whereas in plegic dogs neither a significant difference between the "acute" and "outcome" time points (*p* = 0.6305) nor Treg cell number increase (*p* = 0.60275) were detected.

#### Th17/Treg ratio in dogs with intervertebral disc herniation and study of effects timing, severity, and underlying disease

For Th17/Treg ratio, no statistically significant difference was found for any of the investigated effects of time point, severity, and underlying inflammatory/immunological disease (Figs. 2c, 3c, 4c). The ratios of dogs after recovery of IVDH did not differ significantly from the healthy control population (*p* = 0.8959) (Fig. 5c). At the time point “outcome” two ratios (75.2 and 227.9) differed strongly from the others.

### IL17—measurements in serum samples

In total, IL17 measurement results of 14 dogs were available (53.8%).

In 12 of the recovered dogs (87.7%) IL17 was not detectable. Two dogs (14.3%) showed positive test results. The IL17 values were 659.7 pg/ml and 475.0 pg/ml. One of these dogs had a pre-reported underlying immunological disease. The clinical general examination at the time of the control examination was unremarkable. The other dog had neither underlying disease nor clinical symptoms in the final examination.

## Discussion

Th17 and Treg cells are crucial in the pathogenesis of many inflammatory and immunological responses [18, 37]. In particular, a dysregulated relationship between Th17 and Treg cells was found in many diseases [22–25]. In this prospective study, the involvement and influence of Th17 and Treg cells and the Th17/Treg ratio in the pathogenesis of SCI after IVDH in dogs were investigated. Since SCI may be associated with long rehabilitation periods and potentially permanent neurological deficits, it is important to gain a more detailed understanding of the pathogenesis of SCI in order to identify specific therapeutic strategies [3, 4]. To our knowledge, there has been no comparable study investigating long-term changes in Th17 and Treg cells in human and veterinary medicine in SCI.

Th17 and Treg cell counts were determined in dogs with IVDH after recovery of SCI and compared with corresponding cell counts in the acute stage of the disease and with a healthy control population. The determination of Th17 and Treg cell counts after healing of SCI was necessary to detect whether IVDH leads to cell count changes itself or whether underlying immunological/inflammatory diseases (e.g., atopia) or comorbidities (e.g., bladder infections) are causative for such an imbalance. A baseline determination before the onset of the disease is almost impossible because IVDH is a spontaneous occurring disease and the occurrence cannot be predicted [38]. Therefore, it was necessary to wait a reasonable time for the re-examination to gain a high probability that no further inflammatory reactions in the spinal cord do occur. Studies of dogs that underwent surgery for IVDH showed that clinical improvement is usually completed at six months after surgery [39]. To enhance the probability of completed inflammatory reaction in the spinal cord, the final examinations were chosen at an average of 14 months after the acute stage.

We hypothesized that Th17 and Treg cells are involved in the pathogenesis of SCI after IVDH. In addition, we expected that a coexisting inflammatory/immunological disease leads to an imbalance in the Th17/Treg ratio and that this imbalance would negatively affect the recovery process after SCI.

In accordance with the results of this study, initial low Th17 cell counts in dogs with IVDH were detected by Kämpe et al. [28]. An immediate high consumption of these proinflammatory cells due to increased production and secretion of IL17 was supposed [28]. As a result of high levels of IL17 release, Th17 cells may degenerate, making in vitro staining of these cells impossible [34, 36]. Furthermore, degenerated Th17 cells may not survive the staining process and may no longer be detectable by flow cytometry [34, 36].

Low numbers of Th17 cells were also found in people with chronic back pain, but with concomitant increased numbers of Treg cells [26].

In contrast to the previous described findings, Cheng et al. [27] found significant increased Th17 cell counts, compared to a healthy control population, in 34 human patients with lumbar IVDH [27]. Patients with ruptured discs had higher Th17 and IL17 levels than patients with non-ruptured discs. Rupture of the annulus fibrosus and herniation of nucleus pulposus material have been postulated to trigger an autoimmune response [27]. Shamji et al. [29] also demonstrated elevated IL17 levels in degenerated disc material in humans. Comparisons between the described study and results from human medicine evaluations should be viewed critically. IVDH with chronic disease progression are prominent in humans, whereas in the dog study population, acute disc extrusion was present in 84.6% (*n* = 22) of the examined dogs [27, 40]. There is evidence that acute IVDH initially elicit a stronger immune response and inflammatory reaction than chronic IVDH [41]. Furthermore, the immune response is thought to chronically persist and manifests as chronic pain [27, 41]. In an animal model study, early surgical removal of the nucleus pulposus material was shown to result in decreased pain [42]. In people suffering for five weeks of hyperalgesia, surgery could relieve pain [41]. Luchting et al. [26] argued that chronic pain may lead to immunosuppression and demonstrated increased numbers of Treg cells in human patients with chronic back pain as described above [26]. Therefore, results from studies with acute and chronic IVDH patients are not directly comparable.

In general, it was observed that dogs with coexisting inflammatory/immunological disease had lower Th17 and Treg cell counts than dogs without underlying disease. However, evaluating the two different time points examined, there was no significant difference in the number of Th17 and Treg cells of dogs with and without underlying disease. The number of Th17 and Treg cells increased between the time points regardless of the presence of underlying disease. These results suggest that IVDH caused the initial low Th17 and Treg cell counts that increased to the reference range after recovery from SCI. The coexisting underlying diseases considered in this study were atopy, food allergies and chronic cystitis. In Canine Atopic Dermatitis (CAD), an increased number of Th17 cells was detected in acute skin lesions. In chronic disease stages, fewer Th17 cells were determined [42–44]. A dysregulated immune response in CAD patients is characterized by increased numbers of Th2-, Th17- and Treg cells [43, 45]. Th17 cells are also thought to play a role in pathogenesis in human food allergies by activating Th17 cells and producing IL17 after exposure to an allergen [46, 47].

In human interstitial cystitis, an increasing number of Treg cells with a decreasing number of Th17 cells was recently detected during the course of the disease [48]. In ketamine-induced cystitis, increased Th17 cells were found and an imbalance between Th17 and Treg cells is suspected in the pathogenesis of the disease [49].

According to the studies mentioned above, all the inflammatory/immunological diseases listed can influence Th17 and Treg cell counts. However, in our study, no influence of underlying diseases was detected considering the time points of clinical examinations, although the cell levels tended to be lower than in dogs without concomitant underlying disease. A permanently present, chronically increased consumption of the cells could be causal for this observation. Although we considered different coexisting diseases, comparable findings in the statistical analysis were found. The findings of the whole presented study clearly support the hypothesis that the acute IVDH triggers the change in Th17- and Treg cell counts.

The neurological severity of dogs with IVDH had no statistically significant effect on the number of Th17 and Treg cells in this study. This supports the results from previous studies by Kämpe et al. [28] and Can et al. [50]. More specifically, no statistically significant differences were found when evaluating different degrees of paralysis and when evaluating painful and nonpainful dogs [28, 50]. Kämpe et al. [51] concluded that Th17 cells are not suitable as a prognostic biomarker for SCI [28, 51]. It should be mentioned that older, baseline studies showed that the inflammatory response in the spinal cord is primarily responsible for the development of neuropathic pain and does not correlate with the degree of sensory or motor deficits [41, 52, 53].

In contrast, a positive correlation between increased Th17 cell count and pain intensity was recorded in human patients with disk disease [27]. Animal model studies suggest that T lymphocytes play an important role in the development of neuropathic pain [12, 54–57]. Experimental studies support the involvement of Th17 and IL17 in the development of pain [27, 58]. T helper cells have been detected in peripheral nerves, suggesting that Th17 cells may also contribute to pain development [59]. Furthermore, intrathecal and intraneural injection of recombinant IL-17A triggered hyperalgesia [60].

A balanced ratio between Th17 and Treg cells is important for immune homeostasis and a dysregulated ratio is thought to negatively affect disease progression [18, 37].

In contrast, the current study did not reveal significant differences in the Th17/Treg ratio between time points.

On the one hand, this may indicate the body's ability to maintain immune homeostasis and a balance in the Th17/Treg ratio is maintained by regulating or modulating the number of Th17 or Treg cells accordingly [19]. On the other hand, there were numerous individual differences in the ratios that could have resulted in non-significant differences between the evaluated parameters when statistically analyzed.

Therefore, it cannot be argued that the non-significant differences found in our Th17/Treg ratio are necessarily due to the general ability to achieve immune homeostasis and individual factors could be involved.

In human chronic back pain, an imbalance in the Th17/Treg ratio was found with an increased number of Treg and a decreased number of Th17 cells, which could indicate a link between chronic pain and immunosuppression as mentioned above [26].

A shift in the balance between Th17 and Treg cells in favor of proinflammatory Th17 cells has been detected in several autoimmune diseases, including systemic lupus erythematosus, inflammatory bowel disease, and chronic obstructive pulmonary disease [22–25].

Can et al. [50] could not detect any significant differences in the Th17/Treg ratio at different degrees of paralysis and pain in the acute state of disc disease [50].

Two dogs in our study had a significantly higher Th17/Treg ratio than the rest of the population at the time of the final examination. Both patients had neither a known coexisting underlying disease nor clinical signs in the general examination. In addition, they showed a good course of recovery. Therefore, an acutely present subclinical immunological reaction could have led to the imbalance in the ratio. According to this observation, even a strongly shifted ratio of Th17/Treg does not need to be associated with concurrent clinically visible abnormalities or poor outcome. Based on these aspects, the Th17/Treg ratio cannot be recommended as a prognostic marker in spinal cord injury, as also concluded by Can et al. [50].

Kämpe et al. [28] were able to detect elevated IL17 levels in the acute state of the disease compared to a healthy control population. In a rodent model, maximal IL17 expression was detected 24 h after provoked spinal cord trauma, which continuously decreased during a 72-h measurement period [61]. In this study ELISA was performed at the second time point when no inflammatory response was expected in the spinal cord anymore. The ELISA was negative in 85.7% (*n* = 12), indicating that Th17 cells were not secreting any or very little IL17 after recovery. IL17 was detected in only 14.3% (*n* = 2) of the dogs. The final examination in these two dogs was unremarkable. Food allergy was reported in one of these patients, and no underlying disease was known in the others. A subclinical immunologic reaction present at the time of measurement could be considered as a possible cause. Taking into account the study of Kämpe et al. [28] the results of the ELISAs lead to the conclusion that acute IVDH causes an inflammatory reaction in the spinal cord area, which is accompanied by an increased activation of Th17 cells as well as an increased IL17 secretion [28]. After cessation of the inflammatory reaction in the spinal cord, IL17 is strongly reduced and is not measurable anymore using the described ELISA.

One limiting factor of the study is the relatively small study population. Since our study was a long-term study, dogs were lost during the study period due to a wide variety of reasons such as death (*n* = 13), lack of interest from patient owners (*n* = 15), unavailability (*n* = 2), signs of an acute disease (*n* = 1), or immunosuppressive therapy (*n* = 1). Nevertheless, a sufficiently large group of 26 dogs in total could be analyzed for a successful statistical evaluation.

## Conclusions

Currently, a targeted therapeutic intervention in the Th17/Treg ratio is not feasible. Neither an influence of the underlying immunological disease nor an influence of the neurological severity on the number of Th17, Treg cells, and the Th17/Treg ratio in peripheral blood could be found in recovery state. In addition, there was no association between an imbalance in the Th17/Treg ratio and poor outcome.

Thus, the hypothesis that an altered Th17/Treg ratio after recovery of SCI negatively influences the clinical course could not be confirmed. However, the present findings suggest that altered Th17 and Treg cell levels do occur in the acute stage of IVDH. Only after critical evaluation, they can be considered when evaluating new treatment strategies for the acute stage of the disease.

### Supplementary Information

**Additional file 1.** General data of the study population.

**Additional file 2.** Specific data of the study population.

**Additional file 3.** Descriptive statistical data of Th17-, Treg cell levels and Th17/Treg ratio of the whole dog population.

**Additional file 4.** Descriptive statistical data: difference with and without coexisting underlying inflammatory/immunological disease.

**Additional file 5.** Descriptive statistical data ordered by initial neurological severity.

**Additional file 6.** Measurement results of the study population.

**Additional file 7.** Data of the healthycontrol population.

## Data Availability

The data sets used and/or analyzed in this study are available in the publication as well as in supplementary material. Additional data sets are available upon request from the corresponding author.
