# Correction: Long‑term changes of Th17 and regulatory T cells in peripheral blood of dogs with spinal cord injury after intervertebral disc herniation

**DOI:** 10.1186/s12917-023-03678-1

**Published:** 2023-08-08

**Authors:** M. Wesolowski, P. Can, K. Warzecha, F. Freise, R. Carlson, J. Neßler, A. Tipold

**Affiliations:** 1 Department of Small Animal Medicine and Surgery, University of Veterinary Medicine, Hannover, Germany; 2 Department of Surgery, Faculty of Veterinary Medicine, University of Ankara, Ankara, Turkey; 3 Department of Biometry, Epidemiology and Information Processing, University of Veterinary Medicine, Hannover, Germany

## Correction:

*BMC Vet Res* **19**, 90 (2023)

10.1186/s12917-023-03647-8

Following the publication of the original article [1], it was noted by the author that, due to a typesetting error, all of the texts under the caption of Figs. 1, 2, 3, 4 and 5 were incorrectly placed randomly in text passages in the body text. The misplaced texts should be placed under the figures.

The original article has been corrected.
