# Supplementary material for: Long-term changes of Th17 and regulatory T cells in peripheral blood of dogs with spinal cord injury after intervertebral disc herniation
Source: BMC Vet Res. 2023 Jul 22;19:90. doi: 10.1186/s12917-023-03647-8 (PMC10362779; doi:10.1186/s12917-023-03647-8)
Supplement: Supplementary file 1 — Additional file 1. General data of the study population. [file 12917_2023_3647_MOESM1_ESM.docx]

Additional file 1: General data of the study population

| Dog nummer | Sex | Breed | Age  (in years)  “acute“ | Age  (in years)  “outcome“ | Weight (kg) | Coexist. disease |
| --- | --- | --- | --- | --- | --- | --- |
| 1 | fn | Labrador Retriever | 7 | 9 | 26.0 | food allergy |
| 2 | mn | Bolonka Zwetna | 4 | 5 | 4.2 | - |
| 3 | f | Pug dog | 7 | 8 | 11.0 | - |
| 4 | mn | Maremmano | 5 | 6 | 35.0 | - |
| 5 | f | French Bulldog | 4 | 5 | 12.8 | chronic cystitis |
| 6 | f | Mixed breed | 7 | 9 | 7.6 | atopia |
| 7 | f | Yorkshire Terrier | 6 | 8 | 4.3 | atopia |
| 8 | f | Maltese | 3 | 5 | 4.9 | cystits |
| 9 | mn | Havanese | 8 | 9 | 5.9 | - |
| 10 | m | Dachshund | 2 | 3 | 14.1 | - |
| 11 | fn | French Bulldog | 3 | 4 | 13.4 | atopia |
| 12 | mn | German Sheperd | 7 | 8 | 34.6 | - |
| 13 | m | Mixed breed | 8 | 9 | 29.6 | - |
| 14 | f | Mixed breed | 6 | 8 | 34.4 | - |
| 15 | m | Dachshund | 3 | 5 | 11.2 | - |
| 16 | fn | French Bulldog | 4 | 5 | 12.8 | food allergy |
| 17 | mn | Mixed breed | 5 | 6 | 7.0 | - |
| 18 | m | Jack Russel Terrier | 6 | 8 | 9.4 | - |
| 19 | f | French Bulldog | 3 | 4 | 14.2 | - |
| 20 | mn | Dachshund | 4 | 5 | 3.9 | - |
| 21 | m | French Bulldog | 5 | 6 | 14.4 | - |
| 22 | f | Labrador Retriever | 8 | 9 | 28.0 | - |

m = male; mn = male, neutered; f = female, fn = female, neutered; age in years (acute) = age in the acute stage of

disease before treatment of intervertebral disc herniation (IVDH); age in years (outcome) = age after recovery, on average 14 months after decompressive surgery, coexist. disease = coexisting inflammatory/immunological disease.
