# Supplementary material for: Long-term changes of Th17 and regulatory T cells in peripheral blood of dogs with spinal cord injury after intervertebral disc herniation
Source: BMC Vet Res. 2023 Jul 22;19:90. doi: 10.1186/s12917-023-03647-8 (PMC10362779; doi:10.1186/s12917-023-03647-8)
Supplement: Supplementary file 2 — Additional file 2. Specific data of the study population. [file 12917_2023_3647_MOESM2_ESM.docx]

Additional file 2: Specific data of the study population

| Dog number | Diagnosis | Neurolocalization | Onset | Treatment | Grade (acute) | Grade (outcome) | Intervall  (in month) |
| --- | --- | --- | --- | --- | --- | --- | --- |
| 1 | ANNPE | T3-L3 | acute | conserv. | 3 | 2 | 18 |
| 2 | extrusion | T3-L3 | acute | surg. | 5 | 0 | 16 |
| 3 | extrusion | T3-L3 | chronic | surg. | 2 | 2 | 16 |
| 4 | extrusion | T3-L3 | acute | surg. | 4 | 2 | 15 |
| 5 | extrusion | T3-L3 | acute | surg. | 5 | 3 | 17 |
| 6 | extrusion | T3-L3 | acute | surg. | 2 | 0 | 18 |
| 7 | extrusion | T3-L3 | acute | surg. | 4 | 0 | 15 |
| 8 | extrusion | C1-C5 | acute | surg. | 2 | 2 | 15 |
| 9 | extrusion | T3-L3 | chronic | surg. | 2 | 2 | 16 |
| 10 | extrusion | T3-L3 | acute | surg. | 2 | 0 | 14 |
| 11 | extrusion | L4-S1 | chronic | surg. | 2 | 0 | 15 |
| 12 | extrusion | T3-L3 | acute | surg. | 4 | 2 | 13 |
| 13 | ANNPE | T3-L3 | acute | conserv. | 4 | 2 | 14 |
| 14 | extrusion | L4-S1 | acute | surg. | 3 | 0 | 14 |
| 15 | extrusion | T3-L3 | acute | surg. | 4 | 0 | 15 |
| 16 | extrusion | T3-L3 | acute | surg. | 4 | 0 | 14 |
| 17 | extrusion | T3-L3 | acute | surg. | 5 | 5 | 13 |
| 18 | extrusion | L4-S1 | acute | surg. | 3 | 0 | 14 |
| 19 | extrusion | T3-L3 | acute | surg. | 2 | 0 | 14 |
| 20 | extrusion | T3-L3 | acute | surg. | 2 | 0 | 12 |
| 21 | extrusion | L4-S1 | chronic | surg. | 1 | 0 | 12 |
| 22 | ANNPE | C6-T2 | peracute | conserv. | 3 | 2 | 11 |
| 23 | extrusion | T3-L3 | acute | surg. | 3 | 2 | 12 |
| 24 | extrusion | T3-L3 | acute | surg. | 4 | 2 | 12 |
| 25 | extrusion | T3-L3 | acute | surg. | 2 | 0 | 12 |
| 26 | extrusion | T3-L3 | acute | surg. | 3 | 0 | 11 |

ANNPE = acute non compressive nucleus pulposus extrusion; extrusion = intervertebral disc extrusion.

Neurolocalization: C1-C5; C6-T2; T3-L3; L4-S1 (spinal cord segments: T = thoracal, L= lumbal, S = sacral, C= cervical)

Treatment: conserv. = conservative; surg. = surgery

Grade (acute) = Clinical severity of intervertebral disc herniation (IVDH) according to Sharp and Wheeler (2005) in the acute stage of disease before treatment of IVDH.

Grade (outcome) = Clinical severity of IVDH according to Sharp and Wheeler (2005) after recovery, on average 14 months after decompressive surgery.

Definition of Grade 0-5: 0 = no signs of spinal hyperesthesia or paresis, 1 = spinal hyperesthesia only, 2 = ambulatory paresis, 3 = non ambulatory paresis, 4 = plegia with intact deep pain perception, grade 5 = plegia with loss of deep pain perception

Interval (in month) = Time between the first and final examination.
