# Supplementary material for: Long-term changes of Th17 and regulatory T cells in peripheral blood of dogs with spinal cord injury after intervertebral disc herniation
Source: BMC Vet Res. 2023 Jul 22;19:90. doi: 10.1186/s12917-023-03647-8 (PMC10362779; doi:10.1186/s12917-023-03647-8)
Supplement: Supplementary file 3 — Additional file 3. Descriptive statistical data of Th17-, Treg cell levels and Th17/Treg ratio of the whole dog population. [file 12917_2023_3647_MOESM3_ESM.docx]

Additional file 3: Descriptive statistical data of Th17-, Treg cell levels and Th17/Treg ratio of the whole dog population

| Variable | Median  (cells/μl) | Minimum - Maximum  (cells/μl) | 25% quantile  (cells/μl) | 75%quantile  (cells/μl) |
| --- | --- | --- | --- | --- |
| Th17 (acute) | 20.29 | 2.23 - 107.95 | 9.69 | 41.37 |
| Th17 (outcome) | 42.63 | 0.53 - 124.54 | 33.76 | 69.67 |
| Treg (acute) | 2.15 | 0.16 - 10.47 | 1.20 | 3.29 |
| Treg (outcome) | 7.02 | 0.17 - 28.82 | 2.97 | 10.51 |
| Ratio (acute) | 10.01 | 2.86 - 55.11 | 5.44 | 24.17 |
| Ratio (outcome) | 7.35 | 0.17 - 227.93 | 3.69 | 13.85 |

Data from the study population, consisted of 26 dogs with intervertebral disc herniation (IVDH).

The median, min-max, 25% quantile and 75% quantile for Th17-, Treg cells and the Th17/Treg ratio were given.

“acute” = acute stage of disease before treatment of IVDH; “outcome” = after recovery, on average 14 months after decompressive surgery.
