# Supplementary material for: Long-term changes of Th17 and regulatory T cells in peripheral blood of dogs with spinal cord injury after intervertebral disc herniation
Source: BMC Vet Res. 2023 Jul 22;19:90. doi: 10.1186/s12917-023-03647-8 (PMC10362779; doi:10.1186/s12917-023-03647-8)
Supplement: Supplementary file 4 — Additional file 4. Descriptive statistical data: difference with and without coexisting underlying inflammatory/immunological disease. [file 12917_2023_3647_MOESM4_ESM.docx]

Additional file 4: Descriptive statistical data: difference with and without coexisting underlying inflammatory/immunological disease

| No coexisting disease  (n=19) | Variable | Median  (cells/μl) | Minimum - Maximum  (cells/μl) | 25% quantile  (cells/μl) | 75% quantile  (cells/μl) |
| --- | --- | --- | --- | --- | --- |
|  | **Th17 (acute)** | 21.06 | 8.40 - 107.95 | 12.50 | 60.92 |
|  | **Th17 (outcome)** | 44.47 | 18.06 - 124.54 | 26.12 | 78.15 |
|  | **Treg (acute)** | 2.17 | 0.72 - 10.46 | 19.85 | 3.89 |
|  | **Treg (outcome)** | 8.51 | 0.53- 28.82 | 29.73 | 1.14 |
|  | **Ratio (acute)** | 8.15 | 3.70- 42.19 | 5.12 | 21.95 |
|  | **Ratio (outcome)** | 6.83 | 2.10 - 227.93 | 3.00 | 13.34 |
| Coexisting disease  (n=7) | **Th17 (acute)** | 9.40 | 2.23 - 39.65 | 7.55 | 37.13 |
|  | **Th17 (outcome)** | 38.50 | 2.14 - 65.01 | 3.38 | 58.63 |
|  | **Treg (acute)** | 0.84 | 0.16 - 4.01 | 0.32 | 32.89 |
|  | **Treg (outcome)** | 4.70 | 1.40 - 12.40 | 2.35 | 94.43 |
|  | **Ratio (acute)** | 23.49 | 2.86 - 55.11 | 5.44 | 47.04 |
|  | **Ratio (outcome)** | 9.33 | 0.17 - 25.72 | 4.27 | 23.85 |

“acute” = acute stage of disease before treatment of intervertebral disc herniation (IVDH); “outcome” = after recovery, on average 14 months after decompressive surgery.
