# Supplementary material for: Long-term changes of Th17 and regulatory T cells in peripheral blood of dogs with spinal cord injury after intervertebral disc herniation
Source: BMC Vet Res. 2023 Jul 22;19:90. doi: 10.1186/s12917-023-03647-8 (PMC10362779; doi:10.1186/s12917-023-03647-8)
Supplement: Supplementary file 5 — Additional file 5. Descriptive statistical data ordered by initial neurological severity. [file 12917_2023_3647_MOESM5_ESM.docx]

Additional file 5: Descriptive statistical data ordered by initial neurological severity

| Paresis  (n=16) | Variable | Median  (cells/μl) | Minimum - Maximum  (cells/μl) | 25% quantile  (cells/μl) | 75% quantile  (cells/μl) |
| --- | --- | --- | --- | --- | --- |
|  | **Th17 (acute)** | 16.11 | 2.23 - 107.95 | 92.36 | 45.38 |
|  | **Th17 (outcome)** | 61.82 | 18.06 - 94.72 | 29.93 | 76.24 |
|  | **Treg (acute)** | 2.11 | 0.16 - 28.82 | 0.72 | 35.22 |
|  | **Treg (outcome)** | 8.98 | 2.35 - 9.44 | 4.05 | 10.96 |
|  | **Ratio (acute)** | 9.12 | 3.69 - 55.11 | 5.37 | 25.95 |
|  | **Ratio (outcome)** | 6.30 | 2.10- 31.86 | 3.58 | 12.35 |
| Plegia  (n=10) | **Th17 (acute)** | 25.54 | 8.39 - 91.03 | 17.71 | 41.36 |
|  | **Th17 (outcome)** | 39.14 | 2.14 - 124.54 | 33.91 | 44.47 |
|  | **Treg (acute)** | 2.47 | 0.84 - 4.00 | 1.33 | 3.29 |
|  | **Treg (outcome)** | 4.59 | 0.53 - 14.85 | 1.46 | 9.18 |
|  | **Ratio (acute)** | 11.45 | 2.86 - 47.04 | 5.44 | 24.17 |
|  | **Ratio (outcome)** | 9.62 | 0.17 - 227.93 | 3.69 | 25.72 |

“acute” = acute stage of disease before treatment of intervertebral disc herniation (IVDH); “outcome” = after recovery, on average 14 months after decompressive surgery.
