# Supplementary material for: Long-term changes of Th17 and regulatory T cells in peripheral blood of dogs with spinal cord injury after intervertebral disc herniation
Source: BMC Vet Res. 2023 Jul 22;19:90. doi: 10.1186/s12917-023-03647-8 (PMC10362779; doi:10.1186/s12917-023-03647-8)
Supplement: Supplementary file 6 — Additional file 6. Measurement results of the study population. [file 12917_2023_3647_MOESM6_ESM.docx]

Additional file 6: Measurement results of the study population

| Dog number | Lymphocyte number/μl (acute) | Lymphocyte number/μl (outcome) | Th17 cells/μl (acute) | Th17 cells/μl (outcome) | Treg cells/μl  (acute) | Treg cells/μl  (outcome) | Th17/Treg ratio  (acute) | Th17/Treg  ratio (outcome) |
| --- | --- | --- | --- | --- | --- | --- | --- | --- |
| 1 | 1450 | 1500 | 8.78 | 65.01 | 0.16 | 4.69 | 55.11 | 13.85 |
| 2 | 1550 | 1760 | 19.52 | 33.91 | 3.88 | 9.18 | 5.02 | 3.69 |
| 3 | 1020 | 2400 | 16.63 | 78.15 | 4.42 | 11.42 | 3.76 | 6.84 |
| 4 | 900 | 2110 | 8.39 | 39.77 | 1.28 | 0.53 | 6.56 | 75.22 |
| 5 | 700 | 850 | 21.79 | 38.50 | 4.01 | 1.50 | 5.44 | 25.72 |
| 6 | 510 | 1080 | 7.55 | 55.97 | 0.32 | 2.35 | 23.49 | 23.85 |
| 7 | 1090 | 1300 | 9.40 | 2.14 | 3.29 | 12.40 | 2.86 | 0.17 |
| 8 | 1620 | 2860 | 2.23 | 45.03 | 0.39 | 7.91 | 5.59 | 5.69 |
| 9 | 1410 | 1370 | 11.43 | 24.58 | 0.73 | 8.51 | 15.59 | 2.89 |
| 10 | 1300 | 2870 | 9.70 | 74.33 | 2.62 | 28.82 | 3.68 | 2.58 |
| 11 | 870 | 1970 | 12.50 | 38.45 | 2.10 | 2.88 | 5.95 | 13.34 |
| 12 | 1080 | 1460 | 17.71 | 40.78 | 2.17 | 5.19 | 8.15 | 7.85 |
| 13 | 2190 | 2490 | 29.29 | 124.54 | 1.33 | 0.55 | 21.95 | 227.93 |
| 14 | 970 | 1190 | 15.60 | 18.06 | 2.44 | 3.41 | 6.39 | 5.29 |
| 15 | 1820 | 2220 | 39.65 | 37.09 | 0.843 | 3.98 | 47.04 | 9.33 |
| 16 | 1100 | 800 | 41.36 | 52.30 | 2.80 | 5.28 | 14.76 | 9.90 |
| 17 | 990 | 1820 | 91.03 | 44.47 | 2.16 | 14.85 | 42.19 | 2.99 |
| 18 | 780 | 1430 | 37.13 | 58.63 | 1.20 | 9.44 | 30.85 | 6.21 |
| 19 | 1610 | 3630 | 21.06 | 88.72 | 0.72 | 19.55 | 29.39 | 4.54 |
| 20 | 1100 | 2000 | 53.62 | 26.12 | 10.46 | 12.45 | 5.12 | 2.10 |
| 21 | 1410 | 1810 | 60.92 | 69.67 | 2.14 | 6.14 | 28.40 | 11.35 |
| 22 | 2010 | 1810 | 107.95 | 67.17 | 8.10 | 10.51 | 13.33 | 6.39 |
| 23 | 950 | 1280 | 25.08 | 94.72 | 2.11 | 2.97 | 11.86 | 31.86 |
| 24 | 1450 | 1300 | 34.12 | 23.75 | 2.77 | 1.46 | 9.45 | 16.26 |
| 25 | 1160 | 1470 | 8.46 | 83.41 | 1.98 | 9.99 | 4.26 | 8.35 |
| 26 | 2210 | 3800 | 62.94 | 25.49 | 10.28 | 10.04 | 6.12 | 2.54 |

“acute” = acute stage of disease before treatment of IVDH; “outcome” = after recovery, on average 14 months after decompressive surgery.
