# Supplementary material for: Long-term changes of Th17 and regulatory T cells in peripheral blood of dogs with spinal cord injury after intervertebral disc herniation
Source: BMC Vet Res. 2023 Jul 22;19:90. doi: 10.1186/s12917-023-03647-8 (PMC10362779; doi:10.1186/s12917-023-03647-8)
Supplement: Supplementary file 7 — Additional file 7. Data of the healthycontrol population. [file 12917_2023_3647_MOESM7_ESM.docx]

Additional file 7: Data of the healthy control population

| Dog number | Sex | Breed | Age  (in years) | Lymphocyte number/μl | Th17 cells/μl | Treg cells/μl | Th17/Treg ratio |
| --- | --- | --- | --- | --- | --- | --- | --- |
| 1 | f | Beagle | 4 | 1410 | 22.12 | 8.47 | 2.61 |
| 2 | f | Beagle | 4 | 1550 | 40.88 | 6.26 | 6.53 |
| 3 | f | Beagle | 4 | 2150 | 100.20 | 1.65 | 6.07 |
| 4 | f | Beagle | 4 | 1310 | 38.08 | 2.52 | 15.12 |
| 5 | m | Beagle | 4 | 1750 | 28.08 | 3.42 | 8.22 |
| 6 | m | Beagle | 4 | 1710 | 50.71 | 13.72 | 3.70 |
| 7 | m | Beagle | 4 | 1990 | 15.35 | 2.64 | 5.82 |
| 8 | mn | Beagle | 3 | 1700 | 18.96 | 2.97 | 6.39 |
| 9 | f | Beagle | 1 | 2030 | 26.75 | 2.72 | 9.82 |
| 10 | mn | Beagle | 1 | 2610 | 99.22 | 7.07 | 14.03 |
| 11 | f | Beagle | 1 | 2760 | 52.12 | 12.07 | 4.32 |
| 12 | f | Beagle | 1 | 1640 | 31.98 | 3.45 | 9.28 |
| 13 | f | Beagle | 1 | 1680 | 52.34 | 10.37 | 5.05 |
| 14 | m | Beagle | 1 | 1790 | 35.67 | 4.38 | 8.14 |

m = male; mn = male, neutered; f = female; fn = female, neutered.
